# Supplementary figures and images for: Mast Cells Are Mediators of Fibrosis and Effector Cell Recruitment in Dermal Chronic Graft-vs.-Host Disease
Source: Front Immunol. 2019 Oct 18;10:2470. doi: 10.3389/fimmu.2019.02470 (PMC6813249; doi:10.3389/fimmu.2019.02470)

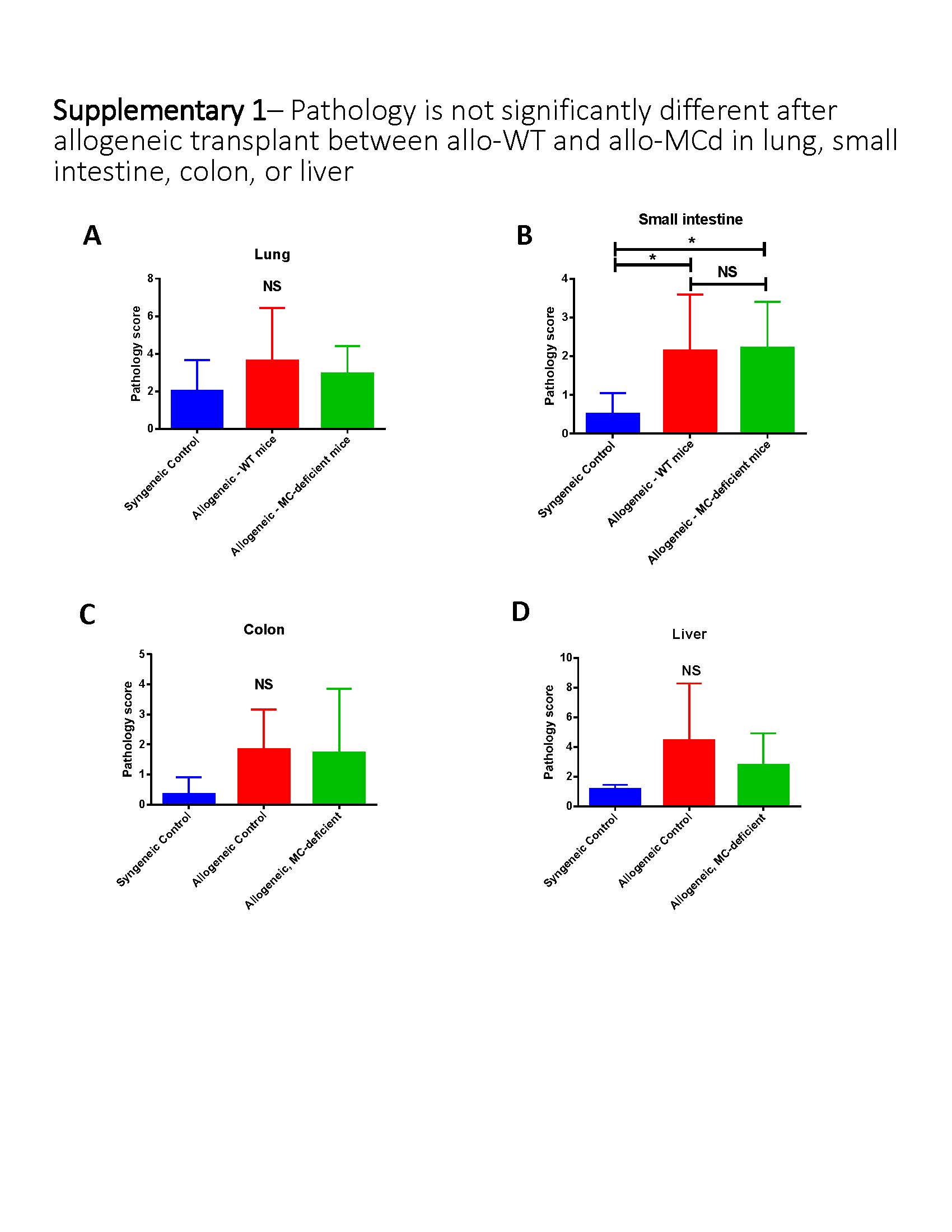

Supplement: Supplementary Figure 1 — Pathology is not significantly different after allogeneic transplant between allo-WT and allo-MCd in lung, small intestine, colon, or liver. Pathology score is unchanged between all groups in (A) lung, (B) small intestine, (C) colon, or (D) liver. Scoring was performed as described previously (18, 54) by a blinded pathologist. Syngeneic (n = 6), allo-WT (n = 16), and allo-MCd (n = 14). Data is combined from two independent transplants. *P = 0.01–0.05, **P = 0.001–0.01, ***P = 0.0001–0.001, ****P < 0.0001, NS, not significant. [file Image_1.JPEG]

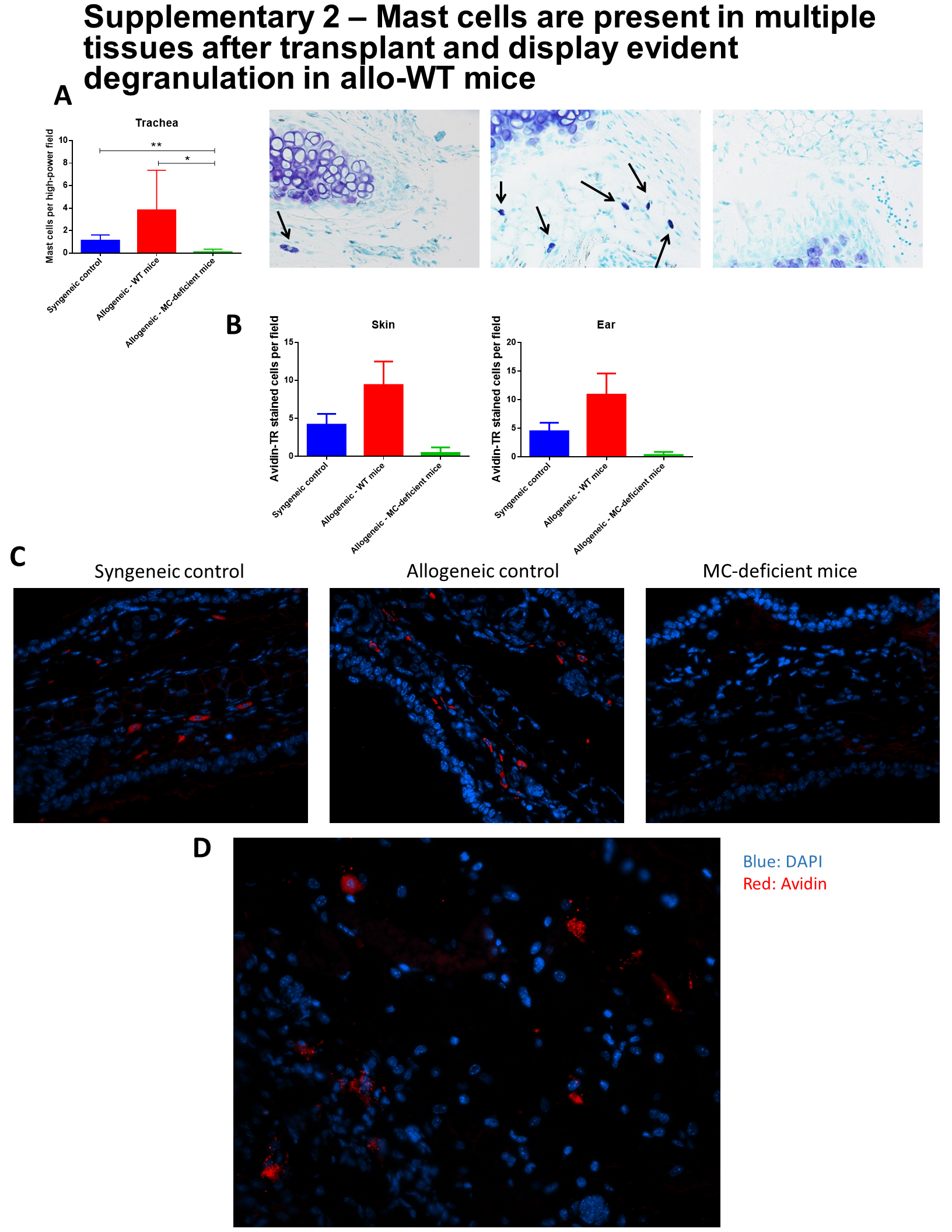

Supplement: Supplementary Figure 2 — Mast cells are present in multiple tissues after transplant and display evident degranulation in allo-WT mice. (A) Mast cell cite counts and toluidine blue-stained representative images in the trachea of syngeneic (n = 6), allo-WT (n = 16), and allo-MCd (n = 14) animals. Mast cell counts per high-power field (400x) are displayed in the first column alongside images from animals whose mast cell count was nearest the mean value for the group. Mast cells (arrows) were denoted by metachromatic staining and granular appearance. (B) Skin and ear sections were stained with avidin and mast cells counted per high-power field (blue = DAPI, red = avidin). (C) Representative images of avidin-stained mast cells in the ear. (D) Degranulation was evident in this representative image of skin from allo-WT mice. *P = 0.01–0.05, **P = 0.001–0.01, ***P = 0.0001–0.001, ****P < 0.0001, NS, not significant. [file Image_2.tif]

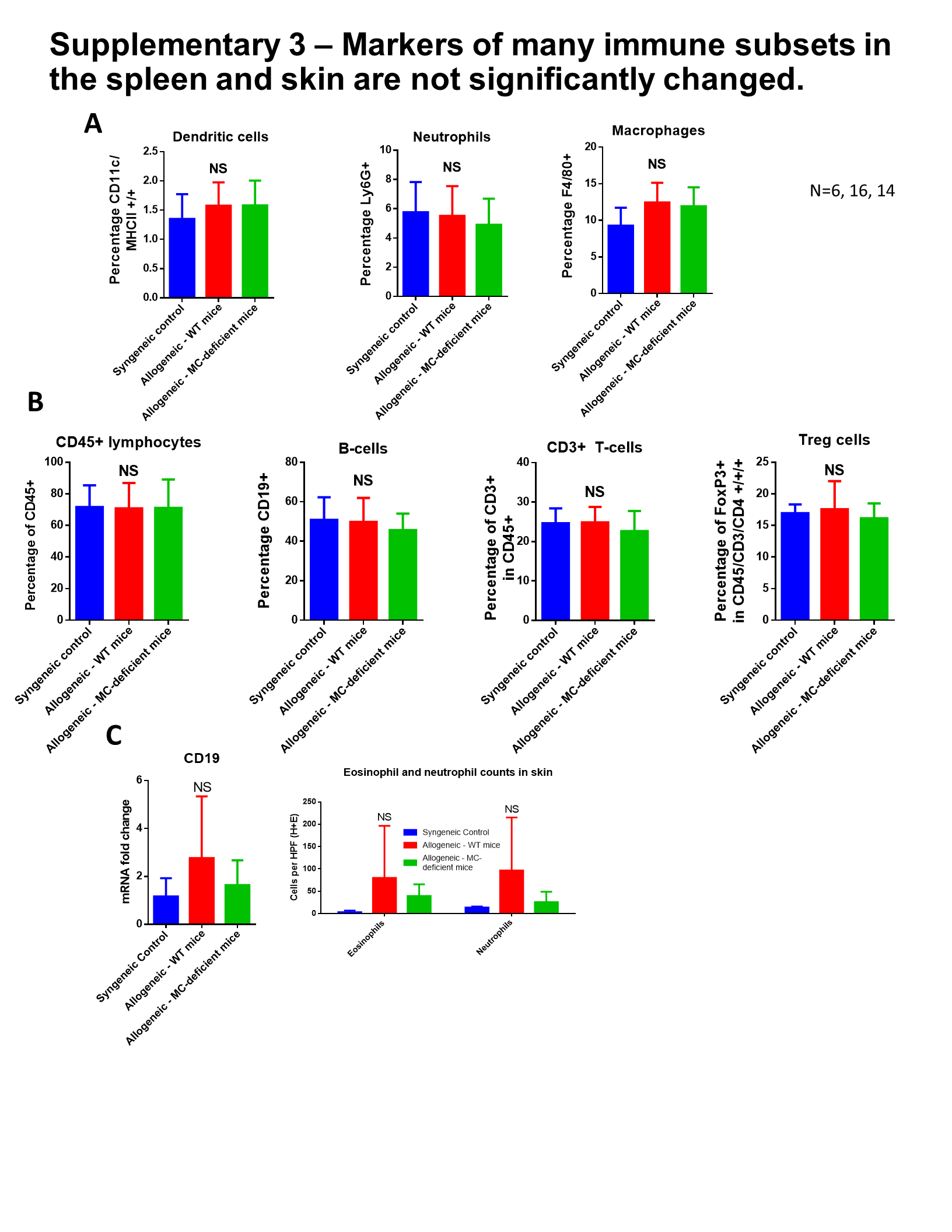

Supplement: Supplementary Figure 3 — Markers of many immune subsets in the spleen and skin are not significantly changed. (A) Myeloid subsets are unchanged in the spleen 7 weeks after allogeneic transplant. MHCII/CD11c+/+ dendritic cells, Ly6G+ neutrophils, or CD11b/F4/80+/+ macrophages have no significant differences in proportion or overall count (data not shown) in the spleen after induction of cGVHD. (B) There were no significant differences in splenic proportion or count (data not shown) of the lymphoid subsets analyzed (CD45+ lymphocytes, CD45/CD19+/+ B-cells, CD45/CD3+/+ T-cells, CD45/CD3/CD4/FoxP3+/+/+/+ T-regulatory cells). This implies that the dermal cGVHD symptomology evident in these mice is driven more strongly by local factors than purely by increased alloreactivity, a conclusion which is consistent with many theories regarding the pathogenesis of fibrotic cGVHD. (C) There is no significant difference in the skin in CD19 transcript (measured by qPCR) or eosinophil/neutrophil counts (counted by a pathologist by H+E morphology). *P = 0.01–0.05, **P = 0.001–0.01, ***P = 0.0001–0.001, ****P < 0.0001, NS, not significant. [file Image_3.tif]

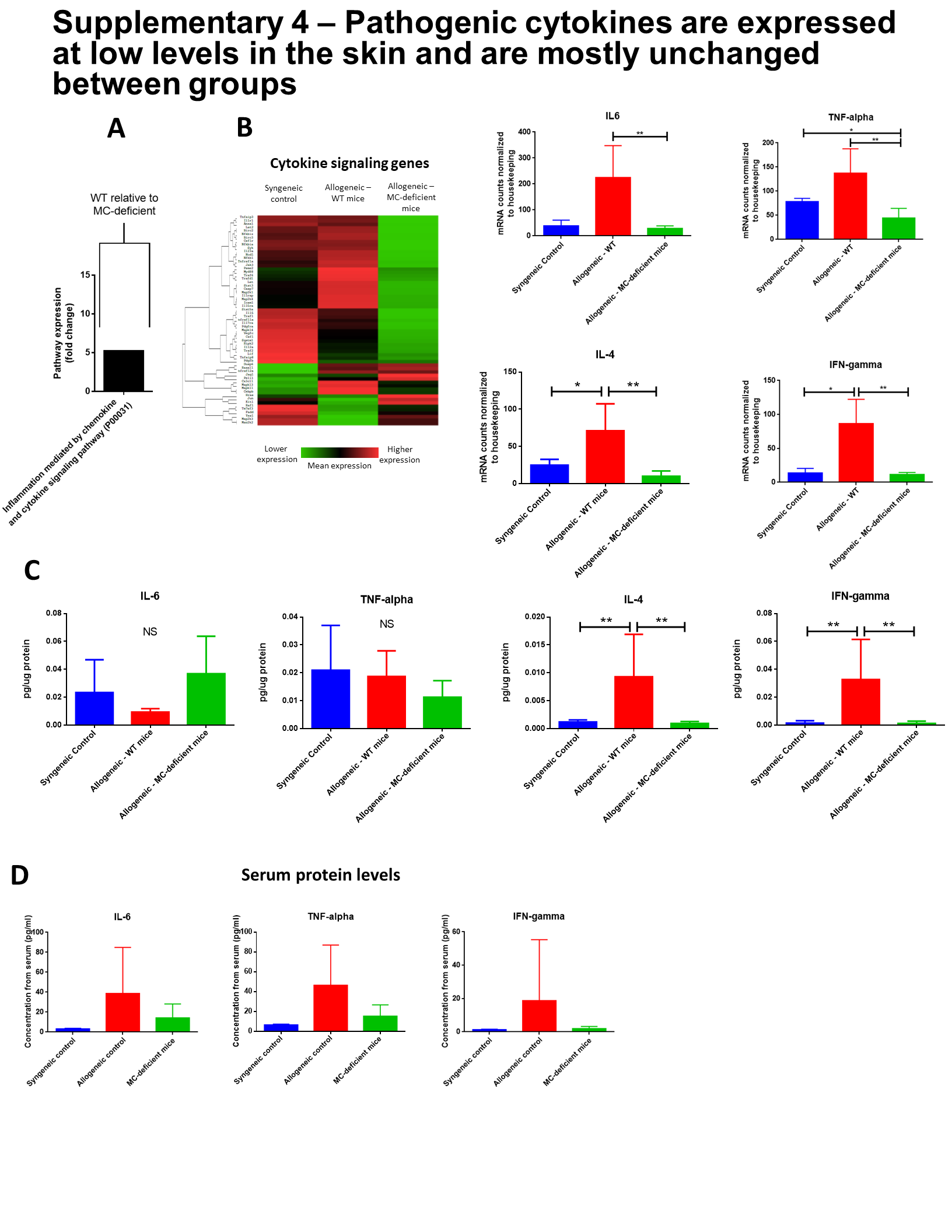

Supplement: Supplementary Figure 4 — Pathogenic cytokines are expressed at low levels in the skin and are largely unchanged between groups. (A) PANTHER pathway analysis demonstrating an increase in genes related to “Inflammation mediated by chemokine and cytokine signaling” in allo-WT relative to allo-MCd. (B) Heatmap analysis and selected genes showing lowered expression of cytokine signaling genes in allo-MCd animals compared to allo-WT animals as measured by NanoString. Heatmaps and gene pathway annotations were generated using NanoString nSolver software. (C) Protein levels were measured in the skin for IL-6, TNF-alpha, IL-4, and IFN-gamma. (D) Protein levels in plasma (syngeneic n = 3, allo-WT n = 8, and allo-MCd n = 7). *P = 0.01–0.05, **P = 0.001–0.01, ***P = 0.0001–0.001, ****P < 0.0001, NS, not significant. [file Image_4.tif]

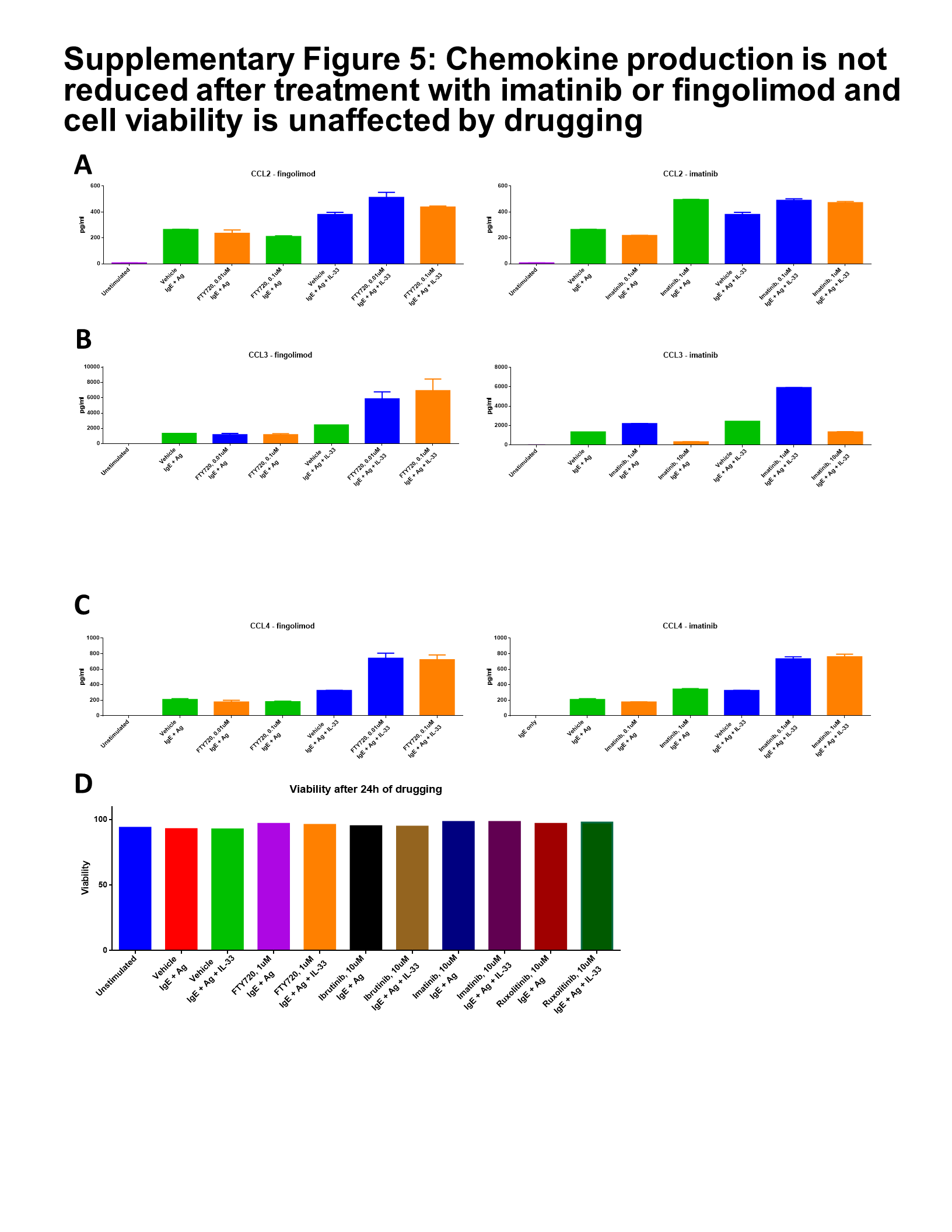

Supplement: Supplementary Figure 5 — Chemokine production is not reduced after treatment with imatinib or fingolimod and cell viability is unaffected by drugging. Mast cells produce high levels of (A) CCL2, (B) CCL3, and (C) CCL4 upon stimulation with IgE + antigen or IgE + antigen + IL-33 (column 1 vs. columns 2 and 6). Production of these chemokines is not decreased by treatment with either imatinib or fingolimod. Results shown are representative of 2–4 independent assays. Error bars are the SD of technical replicates. Chemokine assays were performed using the LEGENDplex Inflammatory Chemokine Assay kit, which measures levels of 13 chemokines. Mast cells did not produce significant amounts of CCL5, CCL11, CCL17, CXCL1, CXCL9, CXCL10, CXCL13, CXCL5, or CCL22 (data not shown). (D) Mast cell viability was unaffected after 24 h of drugging with either imatinib, fingolimod, ibrutinib, or ruxolitinib. *P = 0.01–0.05, **P = 0.001–0.01, ***P = 0.0001–0.001, ****P < 0.0001, NS, not significant. [file Image_5.tif]

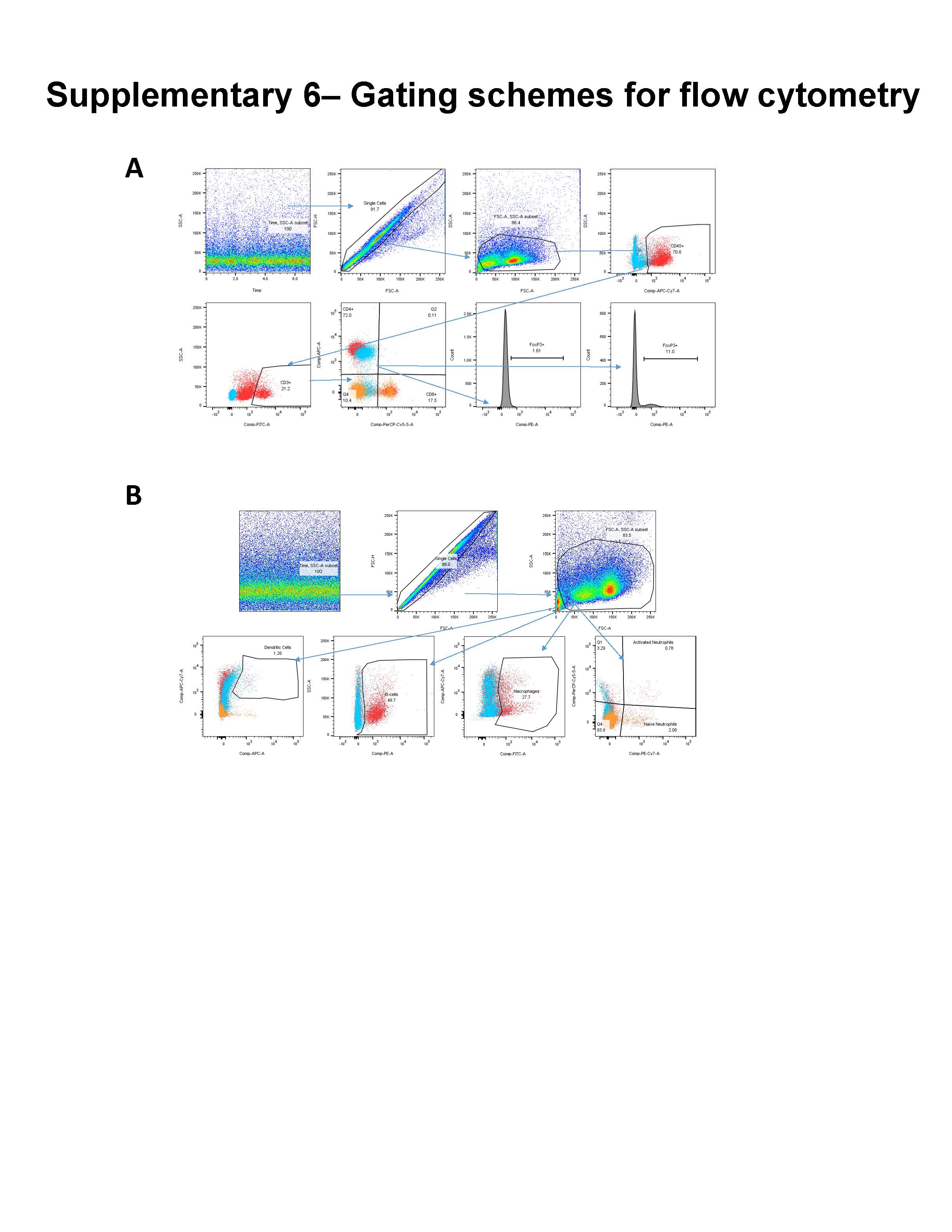

Supplement: Supplementary Figure 6 — Flow cytometry gating schemes. Gating schema for flow cytometry panels run on spleen (Supplementary Figure 3). (A) Gating scheme for a panel to assay T-cell subsets in the spleen. (B) Gating scheme for a panel to assay myeloid subsets and B-cells in the spleen. Red samples are fully stained, while blue, or orange are FMO controls. *P = 0.01–0.05, **P = 0.001–0.01, ***P = 0.0001–0.001, ****P < 0.0001, NS, not significant. [file Image_6.JPEG]
